# The Absorption of the Roots of the Temporary Teeth

**Published:** 1896-08

**Authors:** Allison William Haidle


### The Absorption of the Roots of the Temporary Teeth.

BY ALLISON WILLIAM HAIDLE, D.D.S.

From the earliest period down to the present time many theories have been advanced regarding the phenomenon of the absorption of the roots of the temporary teeth; theories as curious and as varied as may well be imagined, consisting chiefly, however, of mere conjecture, and indeed, at the present time, we can not state positively the whole process in detail.

Many of the ancients supposed that the temporary teeth had no roots. Others thought that the crowns were shed from their roots just as the antlers were shed from the head of a stag, and that the remaining root then gave birth to the permanent tooth.

"Fouchard and Bourdet attributed their removal to the action of a corrosive fluid. Bunon was of the opinion that they were worn away by the rising tooth. Lecluse thought that when the process of their removal began, the blood-vessels ceased to supply nourishing juices, and they were broken up by a species of maceration, while Jordan believed it was both by corrosion and abrasion. Laforge, observing a fungiform or carneous substance behind the root of the temporary tooth (which had, in fact, been noticed by Bourdet, and supposed by him to exude a fluid possessed of solvent qualities), gave it the name of absorbing apparel, and assigned to it the function of removing the root of the temporary tooth. Delabare, who treated the subject at greater length, and apparently investigated it more closely, adopted the views of Laforge."

Harris says the more he has examined the subject the more fully he "has become convinced that it is the result of the action of this fleshy tubercle (the carneous body of Laforge) upon them; and while its formation seems to be the result of the contraction of the dental sac and its appendages for the purpose of effecting the eruption of the permanent tooth, it is especially charged with the removal of everything that would obstruct its passage." Fox attributes it to the pressure of the crowns of the permanent teeth upon the roots of the temporary, but admits, further on, that it frequently occurs without such pressure, and then remarks: "These circumstances seem to prove that the absorption of the fangs of the temporary teeth is an action of nature, sometimes independent of pressure; and it is a very singular circumstance, that at a time of life when so great a quantity of ossific matter is poured forth from all the arteries concerned in the formation of bone, in one particular, there should thus be an absorption of this substance taking place."

Bell rejects the theory of pressure, and attributes the removal to the action of absorbent vessels—he does not state whether veins or lymphatics perform the act.

Nasmyth says: "The functions of the capsular membrane are of great importance at the period when the temporary teeth are removed to make room for the permanent series, inasmuch as it is the agent by which the removal is effected."

McQuillen reasons as follows: "The fact must not be lost sight of, that the dental tissues are but a *congerie* of *cells*, constituting an animal matrix in which the calcareous salts are deposited; that these cells have a definite period of existence, and that by the molecular disintegration they degenerate and are cast off. As the animal matrix degenerates, cell by cell, their calcareous contents are liberated and become mixed with the fluids, to be taken up by the venous or lymphatic radicals. If, in addition to this waste, there is no supply of new material, atrophy is the result, and thus the fangs of the deciduous tooth disappear."

Tomes writes: "Although absorption usually commences on the side of the root which is nearest to the successional tooth, it by no means invariably does so; it may be, and often is, attacked on the opposite side, and in many places at once. The cementum is usually attacked first, but eventually dentine, and even enamel, come to be scooped out and removed by an extension of the process.

"That part of the dentine, however, which immediately surrounds the pulp, appears to have more power of resistance than any other part of the tooth, and thus persists, for a time, as a sort of hollow column. The absorption of the temporary teeth is absolutely independent of pressure; the varying position of the excavation has already been noticed, and it may be added that in many lower animals, for example, the frog or the crocodile, the growing tooth-sac passes bodily into the excavation made before it in the base of the tooth which has preceded it, while if pressure had any share in the matter the cells of its enamel organ, etc., must have inevitably been crushed and destroyed.

"Again, when the absorption and shedding of the first teeth have taken place early, before their successors are ready to appear, perfect little sockets are formed behind the last temporary teeth, cutting them off from the permanent teeth destined to follow them. Absorption, too, may attack the roots of permanent teeth, which is another reason for regarding the process as not necessarily dependent upon approach of a displacing tooth. Closely applied to the excavation produced by absorption is a mass of very vascular soft tissue, the so-called absorbent organ. The surface of this is composed of very large peculiar-looking cells, bearing some little resemblance to those known as 'mycloid cells,' or the 'giant cells' of recent authors. Microscopic examination of the excavated surface shows it to be covered with small hemispherical indentations, the 'lacunæ of Howship,' into each of which one of the 'giant cells' fitted, and in which they may sometimes be seen *in situ*.

"In what manner these 'giant cells' or 'osteoclasts' effect their work is not known, but their presence—where absorption of hard tissues is going on—is universal. Some suppose that they put forth amœbiform processes; others that they secrete an acid fluid, but nothing very definite is known. Abbott does not agree with our English contemporary, and says:

"The assertion of Tomes, that absorption is due to the presence of a freely vascularized papilla, does not explain the decrease of the dental tissues, for the papilla is nothing but medullary tissue, such as we meet with in any part of the organism, where one tissue is about to change into another. Such a papilla may be the cause of the absorption, as well as its result. Another assertion, that the medullary cells eat out the dental tissues by their active growth, or by the amœboid motions, is insufficient to explain the presence of circular or semi-circular excavations and bays, so characteristic of the melting process of the cementum and dentine of deciduous teeth.

"Since we know that pieces of dead bone or ivory may be absorbed with figures similar to those found on the surface of temporary teeth, the idea possibly becomes admissible that, owing to the presence of an acid, first the lime-salts are dissolved away within certain territories of the dead-bone tissue, in a merely chemical or passive manner, whereupon the soft medullary tissue penetrates the spaces thus established. Quite different, however, will be the conception of this process, if we bear in mind that the temporary teeth, as well as the permanent ones, are made up of living tissues, and an active participation of these tissues must be expected in the process of transformation of the dental into the medullary tissue. As the process of absorption is closely allied to the process of inflammation, and active changes of the dental tissues have, beyond any doubt, been proven to follow inflammation, we may, *a priori*, expect such changes of the bone tissue of the temporary teeth in the process of absorption also."

Bodecker quite agrees with Abbott, and says: "The process of absorption of deciduous teeth is, indeed, closely allied to the inflammation process, and may be considered as a physiological type of tissue-changes owing to the presence of some irritating agency. The dissolution of the lime-salts in bone, as well as that of the hard tissues of the teeth, probably is due to the presence of lactic acid. The dissolution of the lime-salts may invade pieces of ivory planted in a living tissue, or invade dead bone tissue. In such dead bones or ivory we likewise observe the disappearance of the lime-salts in the bay-like excavations, which later are filled with a living tissue growing into the excavations from without."

Probably the best conception of what takss place in this process of absorption is that entertained by Dr. G. V. Black. He describes the cellular elements of the peridental membrane as consisting of connective-tissue cells, which are classified as fibro-blasts, cemento-blasts, osteo-blasts and osteo-clasts, classified in this manner according to their size, their shape, and the function they are to perform. It will greatly simplify matters if we bear in mind that each of these cells is but a modified form of the ordinary connective-tissue cell.

With the "fibro-blasts" we need concern ourselves but little; they are such as are destined to reconstruct, or augment in number, the fibers of this membrane. The "cemento-blasts" are also of little moment in this discussion, as they are those which are concerned in the building up or repairing of the cementum. The "osteo-blasts" are polygonal cells which lie upon the surface of the bone, and usually clothe it as epithelium clothes the mucous membranes. They vary so greatly in size that no measurement will give an accurate idea of them. It is very evident from their position that the function of the "osteo-blasts" is the formation of the bone, and there is no growth of bone without their presence. We shall have occasion to refer to them later.

The "osteo-clasts," myoplaxes or giant-cells, Black describes as presenting numerous forms, which vary indefinitely in size, and which are usually multi-nucleated. Occasionally one may be recognized with but a single nucleus, and he has seen them containing as many as twenty-four. From four to ten is a common number. The general inclination is to the round or oblong form. They are very rarely branched, and present no processes. Such forms of cell may be found in other localities, and can be only recognized definitely as "osteo-clasts" when found in contact with bone, or some of the hard tissues undergoing absorption. In such positions they uniformly lie in little bay-like excavations in the surface, known as the "lacunæ of Howship." They conform in certain measure to the depth and size of the excavation in which they lie, which fact seems to argue that most of their growth has occurred in this position. Often very small ones are seen in small excavations, and large ones in correspondingly large excavations. But in absorption of greater extent, such as in the hollowing out of the shafts of the long bones, we often find very large cells lying on the surface of the bone without any lacunæ whatever. The number of such cells, however, that are sometimes seen in the tissue of the bone marrow, detached from the bone, but in the neighborhood of extensive absorption, has given Black the impression that possibly these cells may, in some degree, possess amœboid movement during life, and, therefore, a limited power of migration.

He further says: "The function of these cells is sufficiently obvious; they dissolve the bone with which they are in contact, probably by the secretion of a solvent fluid, making room for themselves, and in this way remove the surface of the bone, *i. e.*, cause its absorption."

To briefly recapitulate: We have now the differentiated connective-tissue cells of the peridental membrane classified—as fibro-blasts, whose function it is to reconstruct the connective-tissue fibers; cemento-blasts, for the building up or repairing of cementum; osteo-blasts, for the formation of bone; and osteo-clasts, for the absorption of the hard tissues.

The most important question that now remains to be solved seems to be: In what manner do the osteo-clasts perform their function?

Very little experimental work has been done to throw light on this vexed question, nor, indeed, are the means at our command to do such work, hence the answers are nearly always mere conjecture. The most popular theory is that an acid is evolved by the osteo-clast, which in turn dissolves the hard tissues, and which is then absorbed and carried away by the lymphatics and blood-vessels. Why this "acid theory" should be so universally accepted the writer is unable to understand, unless it be that analogy is drawn with the hydrochloric acid of the gastric juice, for in no other instance in the animal economy do we find an acid secreted for the performance of a physiological function. Nor is an acid, even in this instance, absolutely necessary for the digestion of proteids. It is well known that comparatively little digestion takes place in the stomach, and that most of the food is ejected in a partially-digested condition into the intestine, where it is acted upon, and even more vigorously than by the gastric juice, by the ferments of the pancreas, the trypsin, the amelopsin, and the steapsin, and this is an *alkaline* medium.

Why is it not more reasonable for us to theorize, that the osteo-clasts act similarly with the cells of the glands which elaborate digestive fluids, that they have a selective influence, and that certain materials are selected from the blood, which unite with other materials present in the cell, that a *ferment* is thus elaborated, and that the roots of the temporary teeth are *digested* rather than dissolved?

It seems to be the tendency of investigators of recent years to ascribe too many and too various functions to the different tissues and organs of the body. When viewed from a physiological standpoint, and when compared to, and brought into relation with, other normal physiological processes, it will be seen that the theory here advanced is quite rational. The chief point for which contention is made, is, that an acid is not necessary for this physiological process, nor, indeed, do acids have the same effect upon teeth as is noticed normally, when subjected to such treatment in laboratory experiments. Nor is a mass of nucleated cells, or a so-called "carneous body" necessary for this process, as is so frequently found described in our literature upon the subject.

Precisely the same cells are concerned, and the same processes are carried on, in the absorption of bone, when a tooth is changed in position in the correction of irregularities, as are concerned and carried on in the absorption of the roots of the temporary teeth. And do we find here a mass of nucleated cells, or a carneous body, or do we think of an acid being secreted for the purpose of dissolving the bone in advance of the approaching tooth?

What probably takes place in the moving of a tooth is, that as pressure is made against it, the osteo-clasts situated in front of the advancing tooth are excited to action, new osteo-clasts are formed from the connective-tissue cells of the peridental membrane, which immediately act in conjunction with those already present, and the bony wall of the alveolus is eaten away or digested—in rare cases only—and then when two or more teeth are involved, is the whole plate of the alveolus moved. As the tooth advances the fibrous tissue behind it is put on the stretch, the osteo-blasts lying against the now deserted wall of the alveolus are also excited to action, their function being to build up and repair bony tissue, and new bone is formed to fill the space.

And in the absorption of the roots of the temporary teeth the osteo-clasts are thrown into functional activity by the pressure of the advancing, permanent tooth, and the roots eaten away or digested and absorbed. There seems to be some doubt in the minds of many as to the part pressure of the permanent tooth plays in absorption, and indeed, Tomes and others assert positively that it plays no part whatever, citing instances where absorption has begun on the side of the root opposite the permanent tooth. It is altogether probable, however, that the osteo-clasts are excited to action reflexly, pressure of the permanent tooth stimulating the sensory nerve-fibers of the peridental membrane, the impulse being reflected back from the central nervous system by way of secretory or trophic fibers, and the cells thus excited.

The fact that absorption may begin on the side of the root opposite the permanent tooth may be explained by the presence of osteo-clasts in this position and their absence in the proper position, their absence being subsequently supplied as necessity and increasing pressure demands.

